# Review of the genus *Promecidia* Lelej, 1996, with description of two new species from China (Hymenoptera, Mutillidae, Trogaspidiini)

**DOI:** 10.3897/zookeys.641.10765

**Published:** 2016-12-16

**Authors:** Arkady S. Lelej, Hu-ting Zhou, Valery M. Loktionov, Zai-fu Xu

**Affiliations:** 1 Institute of Biology and Soil Science, Russian Academy of Sciences, Vladivostok-22 690022, Russia; 2 Department of Entomology, South China Agricultural University, Guangzhou 510640, P. R. China

**Keywords:** Key to species, mutillid wasps, new combination, new species, Oriental, Trogaspidiini

## Abstract

Eleven species of *Promecidia* Lelej, 1996 are reviewed and keyed, and the diagnosis of the genus is given. The genus *Promecidia* is newly recorded from China and *Promecidia abnormis* Lelej, **sp. n.** (China: Guangdong, Hainan) and *Promecidia chui* Lelej & Xu, **sp. n.** (China: Yunnan, Hainan) are described and illustrated. New combination is proposed for *Promecidia boopis* (Kohl, 1882), **comb. n.** (from the genus *Petersenidia* Lelej, 1996). New status is proposed for *Promecidia saturnia* (Mickel, 1935), **stat. n.** and *Promecidia samawangensis* (Mickel, 1935), **stat. n.**

## Introduction

Mutillidae currently include 217 genera and about 4300 described species (Lelej 2007; Lelej and Brothers 2008; Aguiar et al. 2013, updated). In the Palaearctic region 525 species in 61 genera and in the Oriental region 640 species in 64 genera are reported (Lelej 2002, updated; Lelej 2005). The mutillid fauna of China includes 158 species in 32 genera (Chen 1957; Lelej 2002, 2005; He 2004; Tu et al. 2014a, b, 2015), comprising both Palaearctic (northwards of 30°N) and Oriental (southwards of 30°N) taxa. Because of extreme sexual dimorphism, sex associations cannot be made using morphology alone; most species and some genera are known from one sex only. This has resulted in many taxonomic challenges and resulted in many synonyms recognized through matching of males and females.

The genus *Promecidia* Lelej, 1996 was described in the tribe Petersenidiini (as subtribe Petersenidiina) (Lelej 1996) but later when the presumed males of other species were associated and described (Lelej 2005) was placed in the tribe Trogaspidiini. Currently *Promecidia* includes eleven Oriental species which are known from one sex and only *Promecidia saturnia* (Mickel) and *Promecidia chui* Lelej & Xu, sp. n. from both sexes. Both sexes of the genus were keyed as a part of the tribe Trogaspidiini (Lelej 2005). A key to males and females of this genus is given below.

## Materials and methods

The following acronyms are used for the collections where type specimens and other materials are deposited:

**CAS** California Academy of Sciences, San-Francisco, U.S.A.

**HNHM** Hungarian Natural History Museum, Budapest, Hungary.

**IBSS** Institute of Biology and Soil Science, Vladivostok, Russia.

**MRSN** Museo Regionale di Scienze Naturali, Torino, Italy.

**MSNG** Museo Civico di Storia Naturale “G. Doria”, Genoa, Italy.

**NHMW** Naturhistorisches Museum Wien, Vienna, Austria.

**SCAU** Hymenopteran Collection of South China Agricultural University, Guangzhou, China.

**SKYC** Seiki Yamane Collection at Kagoshima University, Kagoshima, Japan.

**USNM** National Museum of Natural History, Smithsonian Institution, Washington, DC, U.S.A.

To study male genitalic characters, the male genitalia were extracted after being previously softened. The muscles were removed in a sodium hydroxide solution (NaOH 10%), several hours without heating; the genitalia were later placed in water to neutralize the NaOH and stored in micro vials filled with glycerin. Male genitalia were studied under a stereomicroscope in a depression slide.

Photographs of imagos and genitalia were taken with a digital camera Cool SNAP attached to Zeiss stereomicroscope Stemi 2000-CS and stacked using CombineZM software (Hadley 2008). The final illustrations were post-processed for contrast and brightness using Adobe® Photoshop® software. The terminology for morphology is based on the glossary provided by the Hymenoptera Anatomy Consortium (2013). The nomenclature of integument sculpture follows Harris (1979), morphological terms are from Brothers (1975) and Lelej (1985). The terminology of wing venation and cells follows Goulet and Huber (1993). Abbreviations are: **POL** postocellar (interocellar) distance between posterior ocelli which is measured dorsally, and **OOL** ocellocular distance between posterior ocellus and compound eye which is measured dorsally.

## Systematics

### Genus Promecidia

Taxon classificationAnimaliaHymenopteraMutillidae

Lelej, 1996

Figs 1–2 , 3–5 , 6–7 , 8–10

Promecidia Lelej, 1996b: 15, ♀; 2005: 80, ♂ & ♀; Lelej and Brothers 2008: 47, ♂ & ♀. Type species: Promecidia yamanei Lelej, 1996, ♀ (Malaysia, Sarawak), by original designation.

#### Gender.

Feminine.

#### Diagnosis.

Male. Head very short, rounded posterad. Eye deeply notched inside. Prementum not tuberculate. Mandible bidentate, with weak subbasal widening beneath or subbasal tubercle (*Promecidia abnormis*, *Promecidia chui*) and without subbasal tooth on inner border (with weak subbasal widening in *Promecidia chui*). Scape curved, rarely widened apically (*Promecidia chui*), with two longitudinal carinae. Ocelli small, POL much shorter than OOL. Tegula not elongated. Mesoscutellum simple, not swollen nor conical. Metacoxa not dentate. Marginal cell of fore wing 1.5× longer than first submarginal cell. Metasomal tergum 2 with lateral felt line. Sternum 2 without lateral felt line. Metasomal sterna 8 (hypopygium) and 7 without strong carina, at most with weak submedian elevation or two submedian carinae on sternum 8 (in *Promecidia chui*, *Promecidia abnormis*) and blunt lateral tubercle on sterum 7 (in *Promecidia abnormis*). Penial valves short, slightly asymmetrical, capitate apically. Volsella with long thin cuspis, stick-like digitus and tuberculate paracuspis. Female. First flagellomere slightly flattened. Anterior part of clypeus with or without two teeth. Mandible slender, with inner preapical tubercle. Scutellar scale lacking. Propodeum dorsally with longitudinal median carina; posterolateral margin of propodeum dentate or serrate. Metasomal tergum 2 with two pale spots located transversely on basal half and with or without pale apical fringe, tergum 3 with pale band. Tergum 6 without distinct pygidial area, convex, smooth, shiny, basal part of tergum punctured, with long pale setae.

**Figures 1–2.**
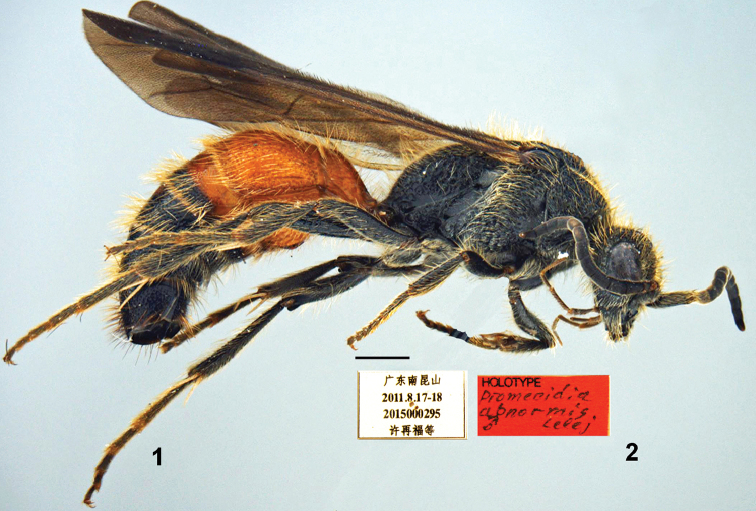
*Promecidia abnormis* Lelej, sp. n., male, holotype. **1** Habitus, lateral view **2** labels. Scale bars 1 mm.

**Figures 3–5.**
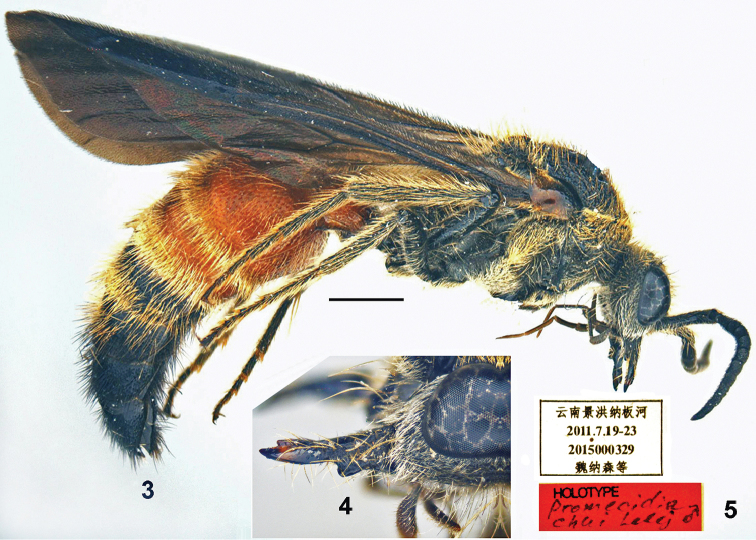
*Promecidia chui* Lelej & Xu, sp. n., male, holotype. **3** Habitus, lateral view **4** mandible, lateral view **5** labels. Scale bars 1 mm.

**Figures 6–7.**
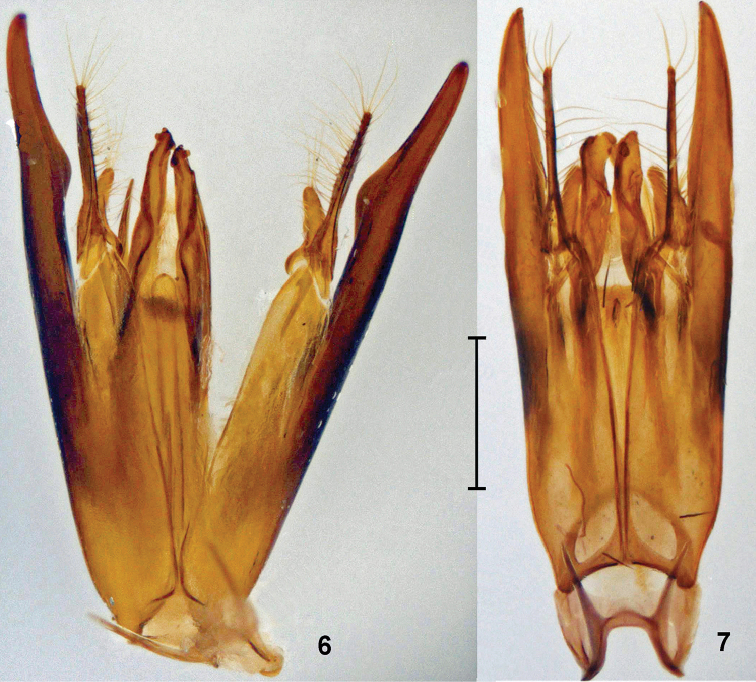
*Promecidia*, male genitalia, ventral view. **6** *Promecidia abnormis* Lelej, sp. n., holotype **7** *Promecidia chui* Lelej & Xu, sp. n., paratype from Hainan. Scale bars 0.5 mm.

**Figures 8–10.**
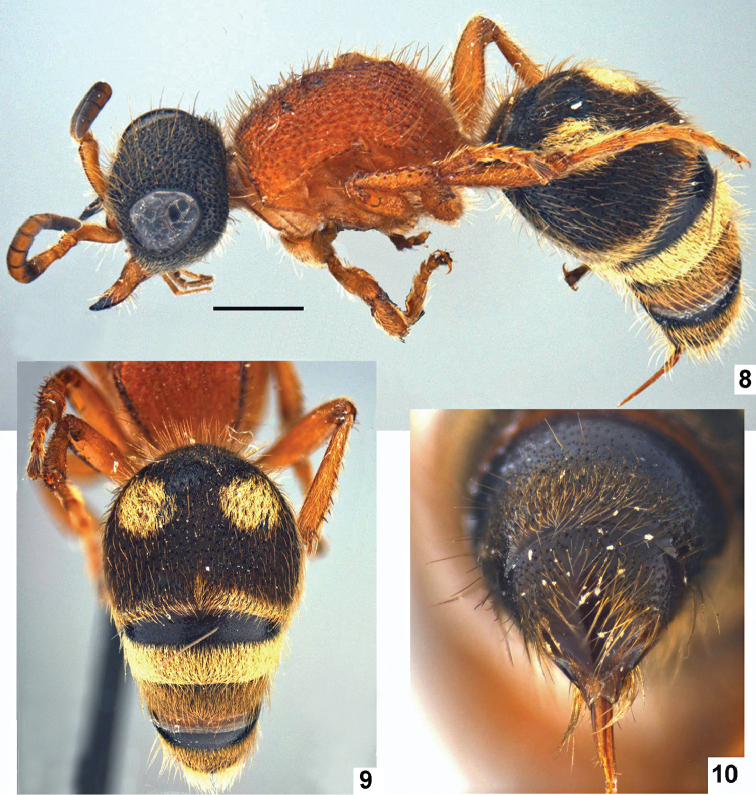
*Promecidia chui* Lelej & Xu, sp. n., female, paratype. **8** Habitus, dorso-lateral view **9** metasoma, dorsal view **10** metasomal terga 5 and 6, dorsal view. Scale bars 1 mm.

#### Species included.

*Promecidia abnormis* Lelej, sp. n., ♂ (China: Guangdong, Hainan); *Promecidia birmanica* (de Dalla Torre, 1897), ♂ (Myanmar); *Promecidia bonthainensis* (André, 1896), ♂ (Indonesia: Sulawesi); *Promecidia boopis* (Kohl, 1882), comb. n., ♀ (Indonesia: Sulawesi); *Promecidia chui* Lelej & Xu, sp. n., ♂ & ♀ (China: Yunnan, Hainan); *Promecidia mamblia* (Cameron, 1902), ♀ (Malaysia: Sabah, Sarawak); *Promecidia ninnii* (Magretti, 1892), ♀ (Myanmar, Vietnam, Laos); *Promecidia rubrocyanea* (Mickel, 1935), ♂ (Malaysia: Sabah); *Promecidia saturnia* (Mickel, 1935), stat. n., ♂ & ♀ (Malaysia: Malay Peninsula; Singapore); *Promecidia samawangensis* (Mickel, 1935), stat. n., ♂ (Malaysia: Sabah, Sarawak); *Promecidia yamanei* Lelej, 1996, ♀ (Malaysia: Sarawak).

#### Sex association.

The male of the type species has not been recognized; the presumed males of other species were associated and described by Lelej 2005: 196. The following pairs of species may eventually be recognized as opposite sexes: *Promecidia yamanei* Lelej, 1996, ♀, and *Promecidia rubrocyanea* (Mickel, 1935), ♂ (both are from Borneo, with dark metallic blue metasoma); *Promecidia boopis* (Kohl, 1882), ♀, and *Promecidia bonthainensis* (André, 1896), ♂ (both are from Sulawesi); *Promecidia mamblia* (Cameron, 1902), ♀, and *Promecidia samawangensis* (Mickel, 1935), ♂ (both are from Sabah and Sarawak); *Promecidia ninnii* (Magretti, 1892), ♀, and *Promecidia birmanica* (de Dalla Torre, 1897), ♂ (both are from Myanmar and Vietnam).

#### Distribution.

Oriental Region.

#### Comments.

The male of *Promecidia* Lelej, 1996 has very short asymmetrical penial valves and definitely belongs to tribe Trogaspidiini. Based on similar penial valves, coupled with having the mandible not strongly excised beneath and simple mesoscutellum the male of *Promecidia* is related to that of Afrotropical *Spinulomutilla* Nonveiller, 1994 but differs by lacking strong lateral carinae on metasomal sterna 7 and 8 (with strong ones in *Spinulomutilla*) and by metacoxa (dentate in *Spinulomutilla*). The male of *Promecidia* is superficially similar with that of *Taiwanomyrme* Tsuneki, 1993 from the tribe Petersenidiini, but differs asymmetrical penial valves (symmetrical in *Taiwanomyrme*). Within the tribes Trogaspidiini and Petersenidiini the female of *Promecidia* easily differs by the absence of a scutellar scale (with more or less developed scutellar scale in other genera of these tribes) and absence of a pygidial area, metasomal tergum 6 glabrous, shiny, not carinate even apically (with more or less developed pygidial area, at least carinate apically in other genera of these tribes; if without pygidial area (*Orientidia* Lelej, 1996) then scutellar scale visible).

#### Key to species of *Promecidia*

**Table:** 

1	Males (unknown in *Promecidia boopis*, *Promecidia mamblia*, *Promecidia ninnii*, *Promecidia yamanei*)	**2**
–	Females (unknown in *Promecidia abnormis*, *Promecidia bonthainensis*, *Promecidia birmanica*, *Promecidia rubrocyanea*, *Promecidia samawangensis*)	**8**
2	Mandible beneath with subbasal denticle; penial valves weakly elongate, apically widened	**3**
–	Mandible beneath basally slightly widened, without subbasal denticle beneath; penial valves shortened, apically capitate	**4**
3	First flagellomere approximately equal in length to flagellomere 2; scape distinctly widened apically; metasomal sternum 8 with weak submedian elevation; sternum 7 without lateral tubercle; sternum 2 with median carina highest basally	***Promecidia chui* Lelej & Xu, sp. n.**
–	First flagellomere 1.4× flagellomere 2; scape not widened apically; metasomal sternum 8 with two submedian carinae; sternum 7 with blunt lateral tubercle; sternum 2 with strong median carina highest apically	***Promecidia abnormis* Lelej, sp. n.**
4	Mesosoma partly or predominantly reddish; metasoma black or dark metallic blue	**5**
–	Mesosoma black; metasoma black with segments 1–3 yellowish-orange	***Promecidia birmanica* (de Dalla Torre)**
5	Metasoma dark metallic blue; metasomal tergum 3 with pale band; mesosoma (except sternum) ferruginous	***Promecidia rubrocyanea* (Mickel)**
–	Metasoma black	**6**
6	Metasomal tergum 3 with pale band; mesosoma mostly black with pronotum, mesonotum and tegula ferruginous	***Promecidia bonthainensis* (André)**
–	Metasomal terga 3–4 with pale band	**7**
7	Mesosoma almost entirely ferruginous with black sterna	***Promecidia saturnia* (Mickel)**
–	Mesopleuron ventrally, propodeum and sterna black, other parts of mesosoma ferruginous	***Promecidia samawangensis* (Mickel)**
8	Metasoma dark metallic blue	***Promecidia yamanei* Lelej**
–	Metasoma black	**9**
9	Metasomal tergum 2 with apical pale fringe or narrow band	**11**
–	Metasomal tergum 2 without apical pale fringe	**10**
10	Humeral angle of mesosoma prominently angulate; mesosoma dorsally with long black setae; larger species: 11.0 mm	***Promecidia mamblia* (Cameron)**
–	Humeral angle of mesosoma angulate but not prominent; mesosoma dorsally with long yellowish setae; smaller species: 5.6–7.6 mm	***Promecidia saturnia* (Mickel)**
11	Posterolateral margin of propodeum dentate; metasomal terga 4 and 5 with black setae	**12**
–	Posterolateral margin of propodeum crenulate; metasomal terga 4 and 5 with rather dense golden setae	***Promecidia chui* Lelej & Xu, sp. n.**
12	Tibiae and tarsi black; pale band on metasomal tergum 3 interrupted medially	***Promecidia boopis* (Kohl)**
–	Tibiae and tarsi ferruginous; pale band on metasomal tergum 3 entire	***Promecidia ninnii* (Magretti)**

### Promecidia abnormis

Taxon classificationAnimaliaHymenopteraMutillidae

Lelej sp. n.

http://zoobank.org/64E4361A-1D5E-42B9-9802-1ECCEF59C72F

Figs 1 , 2 , 6

#### Type material.

Holotype (SCAU). CHINA: ♂, pinned, with genitalia and apical tergum and sternum in a separate micro vial, attached on the same pin, Guangdong, Nankunshan Provincial Nature Reserve, 17–18.VIII.2011, Zai-fu Xu & Hua-yan Chen, No. 2015000295.

#### Paratype.

CHINA: ♂ (SCAU), Hainan, Jianfengling National Nature Reserve, 4–6.V.2008, Bo Qiu.

#### Diagnosis.

Male. Body length 8.5–10.2 mm. First flagellomere 1.2× flagellomere 2. POL:OOL = 0.36. Distance between outer ocellar margins equal to distance between posterior ocelli and posterior border of occiput. Mandible bidentate apically, with subbasal tooth on outer margin beneath. Metasomal sternum 2 (except apical third) with strong longitudinal median carina highest apically. Female unknown.

#### Description.

Male. Body length 8.5–10.2 mm. Black with ferruginous metasomal terga 1–2, sterna 1–3, and base of terga 3 and 4. Head, mandible and scape with yellowish, dense, subappressed setae; pronotum dorsally, mesoscutum, scutellum and metanotum medially with subappressed and erect golden setae, longer on scutellum and metanotum, posterodorsal margin of pronotum densely fringed; propleuron, mesopleuron, and propodeum laterally with whitish appressed and subappressed setae, denser on mesopleuron; posterior propodeal slope with whitish yellow, sparse, erect setae; dorsal propodeal slope with short appressed white setae; legs with dense pale yellow, suberect setae. Metasomal terga with appressed and erect yellowish setae, apically sparsely fringed with pale yellowish setae, sterna with sparse subappressed yellowish setae. Metasomal tergum 2 laterally with felt line, sternum 2 without lateral felt line.

Relation of head width and mesosoma width including tegulae 55:60; relation of maxillary palpus length and cardo length 5.0:1.5. Mandible bidentate apically, with subbasal tooth on outer margin beneath and dorsal carina extending from base to subapical tooth. Clypeus with median area subtriangularly raised, anterior margin with two denticles, distance between them much less than between denticle and base of mandible. Scape not widened apically, bicarinate beneath. First flagellomere 1.2× flagellomere 2; antennal scrobe carinate above. Ocelli small, POL:OOL = 0.36. Distance between outer ocellar margins equal to distance between posterior ocelli and posterior border of occiput. Frons and vertex with shallow dense punctures. Tegula large, not projecting scuto-scutellar suture, with smooth and shiny disc and posterior border. Mesoscutellum evenly convex. Notauli well developed, half length of mesoscutum. Parapsides poorly defined. Metanotum densely punctured, medially with deep glabrous area medially. Pronotum and mesopleuron with large, sometimes confluent punctures; mesoscutum with moderately coarse, more or less separated punctures; mesoscutellum with moderately coarse, somewhat confluent punctures; propleuron obscurely striato-punctate; inferior portions of metapleuron glabrous and shiny. Propodeum reticulate, more finely so laterally, and with larger reticulae dorsomedially.

Fore wing fuscous, first submarginal cell large, subtriangular, apically acute, 0.8× length of marginal cell; second submarginal cell receiving recurrent vein at midpoint; third submarginal cell less distinct than submarginal cell 2 and receiving recurrent vein at midpoint. Pterostigma length equal to distance between origin of *RS_1_* on *SC* and pterostigma.

Carina on metasomal sternum 1 well developed, straight, 0.8× length of sternum 1 in profile, straight; sternum 2 (except apical third) with strong longitudinal median carina highest apically. Terga 1–6 and posterior halves of sterna 3–6 with moderately fine, well separated punctures, larger on tergum 2; sternum 2 with large, moderately coarse, separated punctures; sternum 8 (hypopygium) and tergum 7 (except apical part) with moderately coarse, dense punctures; tergum 7 medially with longitudinal narrow glabrous area up to apical fourth where it with small punctures.

Female. Unknown.

#### Etymology.

The specific name is a Latin adjective meaning “abnormal”, with reference to the unusual carina on metasomal sternum 2, like that of males of *Zeugomutilla* Chen, 1957.

#### Distribution.

China (Guangdong, Hainan).

### Promecidia birmanica

Taxon classificationAnimaliaHymenopteraMutillidae

(de Dalla Torre, 1897)

Mutilla birmanica de Dalla Torre, 1897: 16. New name for Mutilla schlettereri Magretti, 1892.
Mutilla schlettereri Magretti, 1892: 230, tab. 5, fig. 19, ♂, nom. praeocc., *nec* Morawitz, 1890, holotype, ♂, “Monti dei Carin–Chebá, Giugno 1888” [mountain area (1000–1200 m) in the Karen Hills of southeastern Myanmar, in the Tenasserim Mountain Range] (MSNG).
Promecidia birmanica : Lelej 2005: 80, ♂.
Promecidia ninnii : Lelej 2005: 81, part., ♂ (Laos, India: Assam).

#### Material examined.

LAOS: 1 ♂ (MRSN). INDIA: 1 ♂ (CAS), Assam, Kohara, Kaziranga, 110 m, 7.X.1961, E.S. Ross, D.Q. Cavangaro. MYANMAR: 1 ♂ (IBSS), Palaing [16°32'28"N 97°57'34"E], V.1937, R. Perego. VIETNAM: 2 ♂ (IBSS), 70 km NE Saigon (now Ho Chi Minh), Ma de, VIII. 1994, Belyaeva.

#### Diagnosis.

Male. Body length 10.4–12.0 mm (holotype 12.0 mm). Mandible slightly widened beneath at base, without small subbasal denticle. Penial valves shortened, slightly longer than digitus, apically capitate. Mesosoma black. Metasoma black with segments 1–3 yellowish-orange. Female unknown.

#### Distribution.

Myanmar, India, Laos, Vietnam.

#### Comments.

Possibly, this species is the male of *Promecidia ninnii* (Magretti, 1892). Both are distributed in Myanmar and Vietnam.

### Promecidia bonthainensis

Taxon classificationAnimaliaHymenopteraMutillidae

(André, 1896)

Mutilla Bonthainensis André, 1896: 14, ♂, holotype, ♂: “Bonthain” (Indonesia: Sulawesi) (HNHM); Zavattari 1914: 96, ♂.
Timulla (Trogaspidia) bonthainensis : Mickel 1935: 257, ♂.
Promecidia bonthainensis : Lelej 2005: 81, ♂.

#### Material examined.

INDONESIA: Sulawesi, holotype of *Mutilla bonthainensis* André (HNHM), ♂, “781. / 14. // S. Celebes / Bonthain / C. Riobbe 1884 // *Mutilla bonthainensis* / *nensis* / [sic!] det. André // Typus // Holotype / *Mutilla* ♂ / *bonthainensis* André / det. D.J. Brothers 1981 // *Mutilla* / *thoracica* (Smith) / B. Petersen det. 1981”.

#### Diagnosis.

Male. Body length 13.0 mm. Metasoma black. Metasomal tergum 3 with entire pale band. Mesosoma mostly black with pronotum, mesoscutum, mesoscutellum, metanotumn and tegula ferruginous. Female unknown.

#### Distribution.

Indonesia (Sulawesi) (André 1896).

### Promecidia boopis

Taxon classificationAnimaliaHymenopteraMutillidae

(Kohl, 1882) comb. n.

Mutilla boopis Kohl, 1882: 478, ♀, syntypes: “Celebes” (Indonesia) (NHMW); Zavattari 1910: 8, ♀.
Timulla (Trogaspidia) boopis : Mickel 1935: 269, ♀.
Petersenidia boopis : Lelej 2005: 73, ♀.

#### Material examined.

INDONESIA: 1 ♀ (IBSS), Sulawesi, Tomohon, Rurukan, Gn. Mahawu, 1150–1200 m, 30.XI.1999, A. Riedel; 1 ♀ (IBSS), Sulawesi, Kotamobagu, Matalibaru, Torosik, Gn. Tongara, 850–900 m, 9.XII.1999, A. Riedel.

#### Diagnosis.

Male unknown. Female. Body length 6.4–9.0 mm. Metasoma black. Metasomal tergum 2 with apical pale fringe. Posterolateral border of propodeum dentate. Metasomal terga 4 and 5 with black setae. Tibiae and tarsi black. Pale band on metasomal tergum 3 interrupted medially.

#### Distribution.

Indonesia (Sulawesi) (Kohl 1882).

### Promecidia chui

Taxon classificationAnimaliaHymenopteraMutillidae

Lelej & Xu sp. n.

http://zoobank.org/5CE6A276-4F51-47DC-A3CB-031353E076F6

Figs 3–5 , 7 , 8–10

#### Type material.

Holotype (SCAU). CHINA: ♂, pinned, with genitalia in a separate micro vial, attached on the same pin, Yunnan, Nabanhe National Nature Reserve, 19–23.VII.2011, Na-sen Wei & Zai-fu Xu, No. 2015000329.

#### Paratypes.

CHINA: 2 ♂ (SCAU), with the same label as holotype; 3 ♂ (SCAU), Hainan, Bawangling National Nature Reserve, 7–11.VII.2006, Jiang-xian Liu and Li-qiong Weng; 2 ♂ (SCAU), same place, 9–10.VI.2007, Jie Zeng and Bing Xiao; 1 ♂ (SCAU), Wuzhishan Nature Reserve, 16–20.V.2007, Li-qiong Weng; 1 ♂ (IBSS), Jianfengling, 4–7.VI.2007, Jie Zeng; 1 ♀ (SCAU), Diaoluoshan National Nature Reserve, 16–17.VII.2006, Jiang-xian Liu and Li-qiong Weng; 2 ♂ (SCAU), Jiujialing, YPT, 18.VII.2010, Hua-yan Chen.

#### Diagnosis.

Male. Body length 6.8–11.9 mm. First flagellomere equal in length to flagellomere 2. Scape widened apically. Ocelli small, POL:OOL=0.6–0.65. Distance between outer ocellar margins 1.2–1.3× distance between posterior ocelli and posterior border of occiput. Mandible bidentate apically, with subbasal tooth on outer margin beneath, inner margin with weak subbasal widening. Metasomal sternum 2 basally with strong median carina highest basally. Female. Body length 7.65 mm. Mesosoma and legs ferruginous red; flagellomeres 2–9 ferruginous ventrally. First flagellomere 1.33× flagellomere 2. Metasomal tergum 2 with two subcircular spots of yellowish setae anteriorly, separated by distance equal to their diameter. Tergum 6 convex, glabrous and shiny, without definite pygidial area.

#### Description.

Male. Body length 6.8–11.9 mm. Black except ferruginous metasomal terga 1–3 and sterna 1–3; tegula brownish. Frons, vertex, lateral area of clypeus and base of mandible with whitish dense subappressed setae; gena with whitish dense erect setae. Pronotum, propleuron with golden or whitish recumbent setae (posterior margin of pronotum densely fringed); mesonotum with sparse reddish (Yunnan) or white (Hainan) setae. Mesopleuron, lateral area of metanotum and dorsum of propodeum with whitish yellow, dense, appressed setae; posterior propodeal slope with whitish sparse erect setae; mesoscutellum and metanotum medially with erect yellowish setae; legs with yellowish dense suberect setae; lateral propodeal slope with few erect white setae. Metasomal tergum 2 with sparse yellowish setae; terga 3–4 with dense subappressed yellowish setae; terga 1–6 sparsely fringed apically; sterna 1–3 (1–4 in paratypes from Hainan) with sparse subappressed yellowish setae. Metasomal tergum 2 with felt line laterally, sternum 2 without lateral felt line.

Head width 0.84–0.92× mesosoma width including tegulae; relation of maxillary palpus length and cardo length 6.0:1.5. Mandible bidentate apically, with subbasal tooth on outer margin beneath and dorsal carina extending from base to subapical tooth; inner margin weakly widened subbasally. Clypeus subtriangularly raised with median area concave and with anterior margin slightly notched, with transverse preapical carina with few setae. Scape widened apically, bicarinate beneath basally, upper (dorsal) carina complete and widened apically. First flagellomere equal in length to flagellomere 2; antennal scrobe carinate above. Ocelli small, POL:OOL=0.6–0.65. Distance between outer ocellar margins 1.2–1.3× distance between posterior ocelli and posterior border of occiput. Frons, vertex and gena with coarse dense punctures.

Tegula not projecting over scuto-scutellar suture, with smooth and shiny disc and posterior border. Mesoscutellum evenly convex. Metanotum densely punctured, with deep glabrous area medially. Pronotum and mesopleuron with dense, confluent punctures; mesoscutum with dense separated punctures; mesoscutellum with larger coarse, somewhat confluent punctures; propleuron, mesopleuron anterad, metapleuron glabrous. Propodeum reticulate, laterally more finely so, and dorsomedially with larger cells. Notauli well developed, ⅔ length of mesoscutum. Parapsides weaker and shorter than notauli.

Fore wings fuscous, first submarginal cell large, subtriangular, apically acute, 0.7× length of marginal cell; second submarginal cell receiving recurrent vein at midpoint; third submarginal cell less distinct than submarginal cell 2 and receiving recurrent vein at midpoint. Pterostigma length equal to distance between origin of *RS_1_* on *SC* and pterostigma.

Carina on metasomal sternum 1 well developed, 0.8× length of sternum 1 in profile, straight; sternum 2 basally with strong median carina highest basally. Terga 1–6 and posterior halves of sterna 3–6 with moderately fine, well separated punctures, larger on tergum 2; sternum 2 with larger, separated punctures; sternum 8 (hypopygium) and tergum 7 with moderately coarse, dense punctures; tergum 7 medially with narrow glabrous area widened apically.

Female. Body length 7.65 mm. Black with brownish clypeus, palps pale, with ferruginous red mandible (except apex), scape, pedicel, flagellomere 1, legs, mesosoma; flagellomeres 2–9 ventrally ferruginous. Clypeus, gena, mandible with whitish, sparse, erect setae; frons and occiput with subappresseed golden-reddish setae; gena with subappressed whitish setae; dorsum of mesosoma with setae as those on frons; posterior propodeal slope and metasomal tergum 1 with erect whitish setae; metasomal tergum 2 with black dense, recumbent setae; apical fringe of terga 2–3 with yellowish, dense, appressed setae mixed with longer erect ones; terga 4–6 (except pygidial area) with darker and sparser brown setae; sterna 2–6 and legs with yellowish, sparse, erect setae. Metasomal tergum 2 in basal half with two subcircular spots of yellowish setae, separated by distance equal to their diameter; lateral felt line of tergum 2 whitish.

Relation of head width and mesosoma width 47:45 (pronotum): 42 (mesonotum): 37 (propodeum); relation of maxillary palp length and cardo length 3.5:1.5. Head distinctly narrowed behind eyes. Mandible with weak preapical tooth on inner margin. Clypeus posteriorly elevated into rather low, transversely arcuate ridge, with well developed median tubercle basally. First flagellomere 1.33× flagellomere 2; antennal scrobe distinctly carinate above. Eye ovate, strongly convex. Gena not carinate beneath, with tubercle on hypostomal carina closer to mandibular insertion. Frons, vertex and gena with moderately coarse, elongate punctures; vertex with two distinct tubercles located transversely on line of posterior eye borders.

Relation of mesosoma length to its width 55:37, widest on pronotum, narrowest propodeal spiracles. Mesosoma with lateral margins convergent posterad; crenulate, concavely curved, mesoscutum with lateral carina; humeral angles and pronotal lateral tubercle acute, latter prominent. Pleura depressed in center, sharply marked-off from anterior pronotal slope and dorsum of pro- to metanotum by marginal recurvature and from posterior propodeal slope by marginal tubercles; mesopleural suture carinate; dorsal and posterior propodeal slope in profile without definite bend. Dorsum of mesosoma with moderately coarse, deep punctures; posterior propodeal slope with coarser and shallower, elongate, strongly confluent punctures with tendency to rugosity; pleura glabrous.

Legs moderately long; meso- and metatibia each with one row of 5–6 spines and another one with three weak spines; tibio-tarsal relation of hind leg 37:15:11:9:5:7.

Metasomal sternum 1 with anterior arm of Y-shaped ridge moderately strong and posterior ones very weak. Anterior slope of tergum 1 with well separated punctures; dorsal surface of tergum 1 indefinite; tergum 2 with separate setiferous punctures mixed with dense micropunctures; terga 3–5 basally with separate punctures obscured by dense pale setae; tergum 6 (except pygidial area) with dense punctures; sternum 2 basally with median carina, with large separate punctures; posterior areas of sterna 3–6 with fine, separated punctures. Tergum 6 convex, glabrous and shiny, without definite pygidial area; lateral margins not carinate even apically.

#### Etymology.

It is a great pleasure for us (A. Lelej and Z. Xu) to name this species after the well-known Chinese hymenopterist Prof. Chu Joo-tsu (1900–1981).

#### Distribution.

China (Yunnan, Hainan).

#### Comments.

The male and female are collected by the same collectors in July 2006 sites Hainan which are close to each other. There is no direct evidence to support the relationship.

### Promecidia mamblia

Taxon classificationAnimaliaHymenopteraMutillidae

(Cameron, 1902)

Mutilla mamblia Cameron, 1902: 79, ♀, holotype: “Kuching, Sarawak” (Malaysia) (The Natural History Museum, London, UK).
Timulla (Trogaspidia) mamblia : Mickel 1935: 268, ♀.
Petersenidia mamblia : Lelej 1996a: 93, ♀.
Promecidia mamblia : Lelej 2005: 81, ♀.

#### Material examined.

MALAYSIA: 1 ♀ (SKYC), Sabah, Sepilok, forest, 21.VIII.1995, Sk. Yamane; 1 ♀ (IBSS), Sabah, Danum Valley, 30.IV.2000, C. Brühl.

#### Diagnosis.

Male unknown. Female. Body length 9.6–11.0 (holotype) mm. Metasoma black. Metasomal tergum 2 without apical pale fringe. Humeral angle of mesosoma prominently angulate. Mesosoma dorsally with long black setae.

#### Distribution.

Malaysia (Sabah, Sarawak). The record of this species from Perak (Zavattari 1914: 76) is doubtful (Mickel 1935) and probably belongs to a female of *Promecidia saturnia* (Mickel), which is distributed in the Malay Peninsula.

### Promecidia ninnii

Taxon classificationAnimaliaHymenopteraMutillidae

(Magretti, 1892)

Mutilla Ninnii Magretti, 1892: 211, ♀, holotype: “Monti dei Carin–Chebà (900–1100 m. s./m.), Luglio 1888” [mountain area in the Karen Hills of southeastern Myanmar, in the Tenasserim Mountain Range] (MSNG); de Dalla Torre 1897: 66, ♀.
Promecidia ninnii : Lelej 2005: 81, ♀.

#### Material examined.

VIETNAM: 1 ♀ (IBSS), Dong Nai, Ma Da, forest, 6.VI.1995, T. Sergeeva. THAILAND: 1 ♀ (IBSS), North-East Thailand, Nakornratchasima, Sakaerat lowland forest (DEF), 9.VII.1999, Sk. Yamane.

#### Diagnosis.

Male unknown. Female. Body length 6.0 (holotype)–8.4 mm. Metasoma black. Metasomal tergum 2 with narrow apical pale band. Posterolateral border of propodeum dentate. Metasomal terga 4–5 with black setae. Tibiae and tarsi ferruginous. Pale band of tergum 3 entire.

#### Distribution.

Myanmar, Thailand, Vietnam (Dong Nai). The record of this species from India (Assam, Lelej 2005) belongs to *Promecidia birmanica* (de Dalla Torre, 1897) (above).

#### Comments.

Possibly, this species is the female of *Promecidia birmanica* (de Dalla Torre, 1897); both are distributed in Myanmar and Vietnam.

### Promecidia rubrocyanea

Taxon classificationAnimaliaHymenopteraMutillidae

(Mickel, 1935)

Timulla (Trogaspidia) rubrocyanea Mickel, 1935: 256, ♂, holotype, ♂, “British N. Borneo, Bettotan near Sandakan, August 20, 1927, C.B.K. and H.M.P.” [C.B. Kloss and H.M. Pendlebury] (Malaysia: Sabah) (University of Minnesota, St. Paul).
Promecidia rubrocyanea : Lelej 2005: 81, ♂.

#### Material examined.

No specimens examined.

#### Diagnosis

(based on the original description of Mickel 1935). Male. Body length 16.0 mm. Mandible slightly widened beneath at base, without small subbasal denticle beneath. Metasoma dark metallic blue. Mesosoma (except sternum) ferruginous. Metasomal tergum 3 with pale band. Female unknown.

#### Distribution.

Malaysia (Sabah) (Mickel 1935).

### Promecidia samawangensis

Taxon classificationAnimaliaHymenopteraMutillidae

(Mickel, 1935) stat. n.

Timulla (Trogaspidia) saturnia samawangensis Mickel, 1935: 256, ♂, holotype, ♂: “British N. Borneo, Samawang near Sandakan”, July17, 1927, C.B.K. and N.M.P.” [C.B. Kloss and H.M. Pendlebury] (Malaysia: Sabah) (University of Minnesota, St. Paul).
Promecidia saturnia samawangensis : Lelej 2005: 81, ♂.

#### Material examined.

No specimens examined.

#### Diagnosis

(based on the original description of Mickel 1935). Male. Body length 15 mm. Mandible beneath basally slightly widened, without small subbasal denticle beneath. Metasoma black. Metasomal terga 3–4 with pale band. Mesosoma ferruginous except mesopleuron ventrally, propodeum and sterna black. Female unknown.

#### Distribution.

Malaysia (Sabah, Sarawak) (Mickel 1935; Lelej 2005).

#### Remarks.

The males of *Promecidia saturnia* and *Promecidia samawangensis* differ in coloration, distribution and body length; therefore we consider these to be distinct species.

### Promecidia saturnia

Taxon classificationAnimaliaHymenopteraMutillidae

(Mickel, 1935) stat. n.

Mutilla urania Smith, 1857: 83, ♂, *nec* ♀, “Borneo (Sarawak)”.
Timulla (Trogaspidia) saturnia saturnia Mickel, 1935: 256, ♂, holotype, ♂: “Malay Peninsula, Mt. Ophir (labelled as ♂ of Mutilla urania Smith)” (Malaysia: Malacca) (Museum of Natural History, Oxford University).
Promecidia saturnia saturnia : Lelej 2005: 81, ♂.

#### Material examined.

SINGAPORE: 1 ♂ & 1 ♀ (USNM), “Singapore, Coll. Baker” // “K”. MALAYSIA: 1 ♂ (IBSS), “Pasoh Forest Res.[erve] / Negri S.[embilan] Malaysia / x.3.[19]79 sec.[ondary] for.[est] / P. & M. Becker”; 1 ♀ (IBSS), Selangor, Ulu Gombak, VII.1998, F. Ito; 1 ♀ (IBSS), Sabah, Danum Valley, 9.XI.1996, K. Eguchi.

#### Diagnosis.

Male. Body length 10.6–11.7 mm. Mandible beneath basally slightly widened, without small subbasal denticle beneath. Penial valves shortened, slightly longer than digitus, apically capitate. Metasoma black. Metasomal terga 3–4 with pale band. Mesosoma almost entirely ferruginous with black sterna. Female. Body length 5.6–7.6 mm. Metasoma black. Metasomal tergum 2 without apical pale fringe. Humeral angle of mesosoma angulate but not prominent. Mesosoma dorsally with long yellowish setae.

#### Distribution.

Malaysia (Malacca, Negeri Sembilan, Selangor, Sabah), Singapore.

#### Comments.

Possibly the specimens from Singapore are taken *in copula* because they are with the same labels and marked by additional label “K”. The specimens have been collected by C.F. Baker presumably during 1917–1918 when he was Director of Singapore Botanic Gardens (Ascher et al. 2016). The female of *Promecidia saturnia* is related with that of *Promecidia mamblia* (Cameron, 1902). The original description of the both sexes of *Mutilla urania* by Smith (1857) cited Sarawak, Borneo as the type locality. Mickel (1935) examined the syntypes in the Saunders Collection (Museum of Natural History, Oxford University) and found that both syntypes were labelled “Mt. Ophir”, which is located in the Malay Peninsula. The material covered in Smith (1857–1858) paper came from Borneo, Mt. Ophir and Singapore. Possibly, the citation of the type locality as “Borneo” was an error and the correct type locality is Mt. Ophir, Malacca (Mickel 1935). According to Mickel (1935) both sexes of *Mutilla urania* belong to different genera. The female was designated by Mickel as lectotype (holotype *sensu* Mickel 1935) of *urania* which currently belongs to the genus *Odontomutilla* Ashmead, 1899. For the male of *Mutilla urania* (*sensu* Smith 1857) Mickel (1935) proposed a new name Timulla (Trogaspidia) saturnia, but really it was actually a new species based on the paralectotype of *Mutilla urania*, which became the holotype of Timulla (Trogaspidia) saturnia which was later transferred to *Promecidia* Lelej, 1996 by Lelej (2005).

### Promecidia yamanei

Taxon classificationAnimaliaHymenopteraMutillidae

Lelej, 1996

Promecidia yamanei Lelej, 1996b: 15, ♀, holotype, ♀: Malaysia, Sarawak (Malaysia: Sarawak) (SKYC); 2005: 81, ♀.

#### Material examined.

Malaysia: Sarawak, holotype of *Promecidia yamanei* Lelej (SKYC), ♀, “Tower Region / Lambir N. P. / Miri, Sarawak / Malaysia // 17.viii.1995 / Sk. Yamane leg. // Holotype / *Promecidia* / *yamanei* Lelej, ♀”. Other material. MALAYSIA: 1 ♀ (IBSS), Sarawak, Miri, Lambir National Park, Tower Region, 3.III.1997, Sk. Yamane.

#### Diagnosis.

Male unknown. Female. Body length 9.0–12.0 mm. Metasoma dark metallic blue. Head and legs black, mesosoma ferruginous; flagellomeres 2–10 ventrally reddish. Metasomal tergum 2 with two small yellowish spots and narrow apical yellowish fascia interrupted medially, tergum 3 with broad yellowish or golden band.

#### Distribution.

Malaysia (Sarawak).

#### Remark.

Possibly, this species is the female of *Promecidia rubrocyanea* (Mickel, 1935) because both are from Borneo, with dark metallic blue metasoma (other species of *Promecidia* with black metasoma).

## Supplementary Material

XML Treatment for Promecidia

XML Treatment for Promecidia abnormis

XML Treatment for Promecidia birmanica

XML Treatment for Promecidia bonthainensis

XML Treatment for Promecidia boopis

XML Treatment for Promecidia chui

XML Treatment for Promecidia mamblia

XML Treatment for Promecidia ninnii

XML Treatment for Promecidia rubrocyanea

XML Treatment for Promecidia samawangensis

XML Treatment for Promecidia saturnia

XML Treatment for Promecidia yamanei
